# A Germin-Like Protein Gene (*CchGLP*) of *Capsicum chinense* Jacq. Is Induced during Incompatible Interactions and Displays Mn-Superoxide Dismutase Activity

**DOI:** 10.3390/ijms12117301

**Published:** 2011-10-25

**Authors:** Fabiola León-Galván, Ahuizolt de Jesús Joaquín-Ramos, Irineo Torres-Pacheco, Ana P. Barba de la Rosa, Lorenzo Guevara-Olvera, Mario M. González-Chavira, Rosalía V. Ocampo-Velazquez, Enrique Rico-García, Ramón Gerardo Guevara-González

**Affiliations:** 1 División de Ciencias de la Vida, Departamento de Ingeniería en Alimentos, Campus Irapuato-Salamanca, Universidad de Guanajuato, Ex-Hacienda el Copal km 9, Carretera Irapuato-Silao, CP 36500, Irapuato Guanajuato, Mexico; E-Mail: ingfaby@yahoo.com.mx; 2 División de Biología Molecular, Instituto Potosino de Investigación Científica y Tecnológica, Camino a la Presa San José 2055, Colima, Lomas 4 sección CP 78216, San Luis Potosí, S.L.P. Mexico; E-Mails: ahuizolt_j@hotmail.com (A.J.J.-R.); apbarba@ipicyt.edu.mx (A.P.B.R.); 3 Laboratorio de Biología Molecular, Departamento de Ingeniería Bioquímica, Instituto Tecnológico de Celaya, Avenida Tecnológico y A, García-Cubas, S/N, Col, FOVISSSTE, CP 38010, Celaya, Guanajuato, Mexico; E-Mail: lorenzogo@yahoo.com; 4 C.A de Ingeniería de Biosistemas, Facultad de Ingeniería, Universidad Autónoma de Querétaro, Centro Universitario Cerro de las Campanas, S/N, Col, Las Campanas, CP 76010, Santiago de Querétaro, Querétaro, Mexico; E-Mails: torresirineo@gmail.com (I.T.-P.); rosov05@yahoo.com.mx (R.V.O.-V.); ricog@uaq.mx (E.R.-G.); 5 Unidad de Biotecnología, Instituto Nacional de Investigaciones Forestales, Agrícolas y Pecuarias, Carr, Celaya-San Miguel de Allende, km 6, CP 38010, Celaya, Guanajuato, Mexico; E-Mail: gonzalez.mario@inifap.gob.mx

**Keywords:** GLP, geminiviruses, Mn-SOD, ethylene, salicylic acid

## Abstract

A germin-like gene (*CchGLP*) cloned from geminivirus-resistant pepper (*Capsicum chinense* Jacq. Line BG-3821) was characterized and the enzymatic activity of the expressed protein analyzed. The predicted protein consists of 203 amino acids, similar to other germin-like proteins. A highly conserved cupin domain and typical germin boxes, one of them containing three histidines and one glutamate, are also present in CchGLP. A signal peptide was predicted in the first 18 *N*-terminal amino acids, as well as one putative *N*-glycosylation site from residues 44–47. *CchGLP* was expressed in *E. coli* and the recombinant protein displayed manganese superoxide dismutase (Mn-SOD) activity. Molecular analysis showed that *CchGLP* is present in one copy in the *C. chinense* Jacq. genome and was induced in plants by ethylene (Et) and salicylic acid (SA) but not jasmonic acid (JA) applications in the absence of pathogens. Meanwhile, incompatible interactions with either *Pepper golden mosaic virus* (PepGMV) or *Pepper huasteco yellow vein virus* (PHYVV) caused local and systemic *CchGLP* induction in these geminivirus-resistant plants, but not in a susceptible accession. Compatible interactions with PHYVV, PepGMV and oomycete *Phytophthora capsici* did not induce *CchGLP* expression. Thus, these results indicate that *CchGLP* encodes a Mn-SOD, which is induced in the *C. chinense* geminivirus-resistant line BG-3821, likely using SA and Et signaling pathways during incompatible interactions with geminiviruses PepGMV and PHYVV.

## 1. Introduction

Germins and germin-like proteins (GLPs) have been implicated in the reinforcement of plant cell wall strength providing plants with resistance to biotic and abiotic stresses [1–3]. A key feature of the GLP-related subfamilies, including germins, is the presence of germin boxes and conservation of a cupin superfamily (beta-barrel proteins) derived-motif [4–7]. This motif contains three highly conserved histidines and one glutamate residue involved in binding a metal ion [8]. GLPs have been classified into subfamilies based on having oxalate oxidase (OXO) or superoxide dismutase (SOD) activities [7–10]. OXO and SOD activities result in production of hydrogen peroxide. A new GLP gene (hereafter named as *CchGLP*) was previously identified in a geminivirus-resistant *Capsicum chinense* line named BG-3821 [11,12]. *CchGLP* is specifically induced in incompatible interactions of *C. chinense* BG-3821 with *pepper huasteco yellow vein virus* (*PHYVV*), *Xanthomonas campestris* pv. vesicatoria and *Fusarium solani* [11]. According to classification suggested by Park *et al*. [2] and based on this expression pattern in different plant-microbe interactions, *CchGLP* was considered a pathogenesis-related (PR) protein of the family PR-16 [11]. PR proteins are defined as host plant proteins induced specifically in pathological or related situations. They are not accumulated locally in the infected leaf, but are also systemically induced, associated with the development of systemic acquired resistance (SAR) against further infection by fungi, bacteria and viruses [13]. In order to characterize in more detail the possible role of *CchGLP* in pathogen resistance in *C. chinense* BG-3821, it is necessary to evaluate gene expression in other plant-pathogen interactions as well as molecular aspects of this gene and the functional nature of the protein. Therefore, the aim of this work was to characterize the *CchGLP* gene at the molecular level and the enzymatic activity of the expressed recombinant protein, as well as to evaluate *CchGLP* expression in different compatible and incompatible interactions. The deduced amino acid sequence of the *CchGLP* gene contains a signal peptide, conserved histidines and glutamate residues as well as a germin boxes, as reported for other GLPs. Moreover, a manganese superoxide dismutase (Mn-SOD) activity for the *CchGLP* protein expressed in *E. coli* was detected. Molecular studies revealed that *CchGLP* is present in one copy in the genome. In addition, *CchGLP* was locally and systemically induced during incompatible interactions with *pepper golden mosaic virus* (PepGMV*)* and after ethylene and salicylic acid applications in the absence of pathogen in this geminivirus-resistant line. Several implications of our results regarding the plant-microbe interactions are discussed.

## 2. Results and Discussion

### 2.1. Characterization of the *CchGLP* Sequence

**Figure 1:**
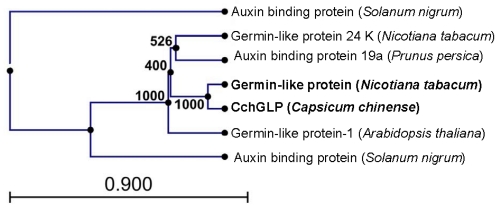
Phylogenetic tree of *CchGLP* amino acid sequences and other plant GLPs. Accession numbers of GLP amino acid sequences evaluated in the tree are: *C chinense* (ABG76199), *A. thaliana* (NP_177405), Auxin binding protein from *Solanum nigrum* (ADW66138), GLP 24 K from *Nicotiana tabacum* (BAC77634), *GLP* from *Nicotiana tabacum* (BAH15357), *Auxin binding protein* from *Solanum nigrum* (ADW66150) and auxin binding protein 19a from *Prunus persica* (Q9ZRA4).

The *CchGLP* cDNA sequence contains a 24 bp leader section, poly A tail, and an ORF encoding a predicted protein of 203 amino acids. Figure 1 shows a phylogenetic tree of amino acid sequences from several solanaceae and *Arabidopsis thaliana* plant proteins, which according to BLAST analysis were the most related to *CchGLP.* The deduced protein of *CchGLP* was more related to a GLP of *Nicotiana tabacum* (93% identity, accession number: BAH15357).

**Figure 2:**
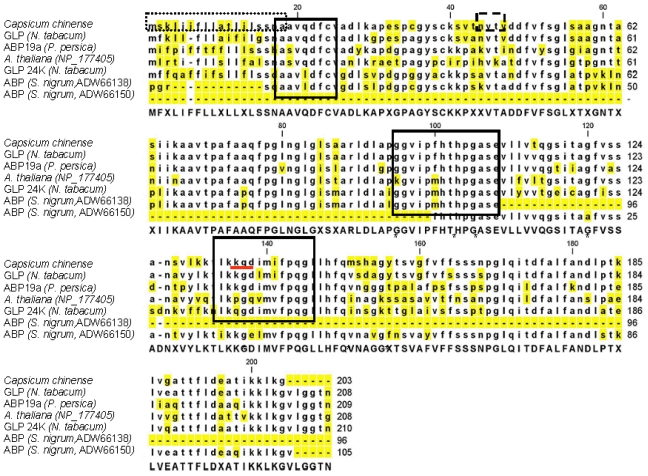
Amino acid sequence alignment of CchGLP and GLPs from some *Solanaceae* and *A. thaliana*. Amino acids forming the germin boxes are indicated with solid squares. The long dashed rectangle in the *N*-terminus indicates amino acids of predicted signal peptide. The short dashed rectangle shows a predicted *N*-glycosylation site (NVTV). The red line shows the tripeptide KGD (or sometimes KGE), characteristic of almost 50% of GLPs. Asterisk display important invariable amino acids that are conserved in GLPs. Yellow highlighting displays conserved amino acid residues among aligned GLPs.

The *CchGLP* protein sequence was submitted to bioinformatic analysis in order to characterize it in more detail. A highly conserved cupin 2 domain (not shown) and structure that contains three boxes representing the germin domains according to Lu *et al*. [14] are present in *CchGLP* (Figure 2). In addition, *CchGLP* was predicted to contain a *N*-terminal signal peptide of 18 amino acids, and one potential *N*-glycosylation site from amino acids 44–47 (NVTV) (Figure 2). Moreover, a putatively important sequence KGD (or sometimes KGE) that has been detected in over 50% of GLPs, but not in germins was detected in *CchGLP* (Figure 2).

The number of amino acids in the deduced protein as well as sequence features were typical of those reported in plant GLPs elsewhere [2,4,6]. Glycosilation in germins appears to be essential in protein-protein interactions but not for enzyme activity as demonstrated by Pan *et al*. [15].

### 2.2. *CchGLP* Protein Purification

**Figure 3:**
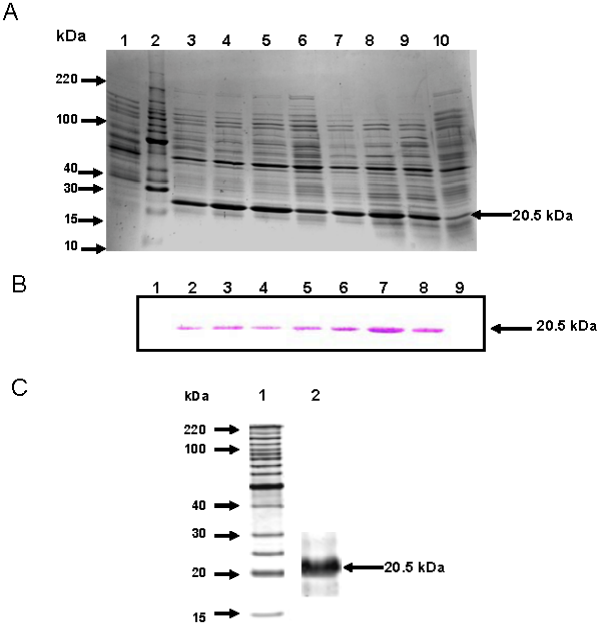
*In vitro CchGLP* protein production in *E. coli*. Panel A, SDS-PAGE displaying *CchGLP* (Germin-Like protein) produced during 24 h post-IPTG induction. Lane 1, negative control [proteins extracted from *E. coli* transformed with plasmid pET22b (+)]; Lane 2, molecular size marker; Lanes 3–10, proteins obtained in *E. coli* transformed with plasmid pET22b (+)-*CchGLP* at 4, 7, 10, 13, 16, 19, 24 and 0 h post-IPTG induction. Panel B, Western blot analysis for recombinant protein production in *E. coli* transformed with plasmids harboring or not the full-length *CchGLP* cDNA sequence. Lane 1, negative control; Lanes 2–9, protein extracts from *E. coli* at 24, 19, 16, 13, 10, 7, 4 and 0 h post-IPTG induction. Antibody used was specific against the His tail engineered in the recombinant protein (see Materials and Methods). Panel C, Lane 1 molecular size marker; Lane 2, *CchGLP* (germin-like protein) recombinant protein purified using Ni-column. In all panels, arrows indicate the presence of *CchGLP*. Molecular size marker used was BenchmarkTM (Invitrogen).

To carry out a more detailed analysis of *CchGLP*, the recombinant protein was produced in the *E. coli* system. A protein with the expected molecular weight for *CchGLP* protein without the signal peptide (20.5 kDa) was mainly detected in the soluble fraction (over 20% of total proteins). The recombinant protein was observed 4 h after IPTG induction, but was not observed in the control test without induction (Figure 3A). Recombinant *CchGLP* protein was further verified with Western blot analysis using specific antibodies against the His-tail tag added to the protein (Figure 3B). After purification of the recombinant protein through Ni-columns, the elution fractions showed a band of 20.5 kDa with a purity of above 95% (Figure 3C). This purified protein was used for enzymatic activity determinations. It is worth mentioning that in contrast to reported problems of expression of germin proteins in *E. coli* due to protein aggregation [16], in this work we did not have these problems that appeared to reduce expression in this system.

### 2.3. In Vitro Enzymatic Activity of *CchGLP*

**Figure 4:**
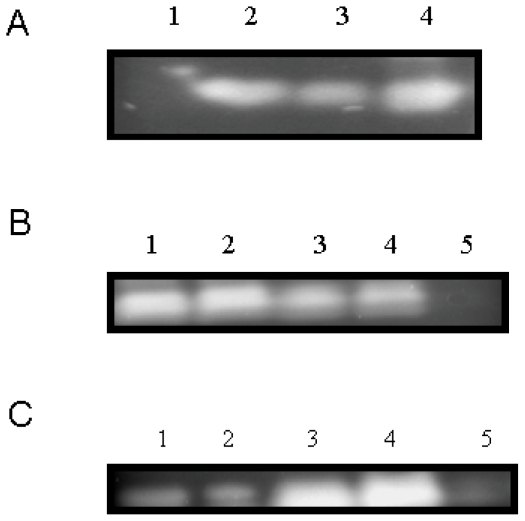
Enzymatic activity of *CchGLP* recombinant protein. Panel A, SOD activity determination. Lane 1, negative control (25 μg of purified tannase from *A. niger*); Lanes 2 and 3, 25 and 15 μg of purified *CchGLP* protein, respectively; Lane 4, bovine erythrocyte superoxide dismutase (15 μg). Panel B, Effect of H_2_O_2_ on *CchGLP* activity. Lanes 1–4, 25, 20, 15 and 10 μg of purified *CchGLP* protein; Lane 5, positive control (10 IU of commercial Fe-SOD from *E. coli*). Panel C, Effect of KCN on *CchGLP* activity. Lanes 1–4, 10, 15, 20 and 25 μg of purified *CchGLP* protein; Lane 5, positive control (10 IU of bovine erythrocyte Cu/Zn-SOD).

Proteins belonging to the group of “germins” show an OXO activity mainly related to their role in defense [2]. On the other hand, “germin-like” proteins can also accumulate during pathogen attack although they are totally devoid of OXO but display SOD activity [2]. Moreover, several germin proteins expressed in *Pichia pastoris* might form multimers which are required for activity [15] and homohexameric barley germin, for which the 3D structure has been solved, displays both OXO and SOD activities [17,18]. Thus, in order to determine whether CchGLP displayed either OXO or SOD activity (or both activities), these assays were carried out. OXO activity was not detected for *CchGLP* (data not shown), instead the *CchGLP* purified protein displayed SOD activity (Figure 4A). Inhibition assays of SOD activity using either H_2_O_2_ (inhibits Fe-SOD activity) or KCN (inhibits Cu/Zn-SOD activity) are used to identify the type of SOD activity [19]. On the other hand, Mn-SOD activities are resistant to both compounds [19]. Thus, inhibition assays of SOD activity revealed that CchGLP was not inhibited by either compound, indicating Mn-SOD activity for this protein (Figure 4B,C).

Plant Mn-SODs have been reported to occur extracellularly as well as in mitochondria and peroxisomes [5,18]. Moreover, Mn-SOD activities have previously been associated with defense against biotic stress in plants [13,18].

### 2.4. Molecular Characterization and Gene Expression of *CchGLP* during Plant-Microbe Interactions

**Figure 5:**
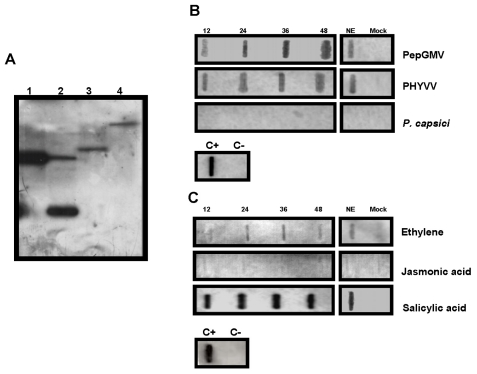
Determination of genomic copy number and expression profile with several inducers of *CchGLP*. Panel A, *C. chinense* BG-3821 genomic DNA was digested with either *Pst* I (cut inside *CchGLP* gene), *Hind* III (cut outside *CchGLP* gene) or *Eco* RI (cut outside *CchGLP* gene) enzymes. Lane 1, positive control (5 μg of linearized plasmid PCR4.0-*CchGLP Eco* RI digested); Lanes 2–4 signal displayed of 20 μg of *C. chinense* BG-3821 genomic DNA digested with either, *Pst* I, *Hind* III and *Eco* RI, respectively. Panel B, *CchGLP* gene induction in *C. chinense* BG-3821 inoculated leaves after 12, 24, 36 and 48 h post-infection; NE, newly emerged leaf; Mock, mock-inoculated leaf with plasmid bluescript SK+ (geminivirus) or uninoculated potato dextrose agar blocks (*P. capsici*). Panel C, the same order of lanes as in panel B, but using plant growth regulators in the absence of geminiviruses or oomycetes as possible *CchGLP* inducers. Mock-inoculation was carried out with deionized water. In all slots, 15 μg of total RNA were loaded. The complete ORF sequence of *CchGLP* was used as a probe in all cases. C− and C+, are negative and positive controls of hybridization, using *C. annuum* genomic DNA and plasmid PCR4.0-*CchGLP*, respectively.

The *CchGLP* cDNA sequence was compared with a genomic sequence obtained using PCR with specific oligonucleotides. No differences were detected between the sequences, indicating no introns are present in the *CchGLP* genomic sequence. Hybridization assays using total genomic DNA from *C. chinense* BG-3821 (geminivirus-resistant) and UX-SMH-55 (geminivirus-susceptible, data not shown), showed either a single or two bands when genomic DNA was digested with restriction enzymes cutting outside or once inside the *CchGLP* gene, respectively (Figure 5A). *CchGLP* was not detected in hybridization assays with *Capsicum annuum* cv. “Sonora Anaheim” genomic DNA (data not shown). These results indicated a single copy of the *CchGLP* gene in both *C. chinense* accessions, and the absence of the *CchGLP* gene in *C. annuum*. On the other hand, *CchGLP* expression was studied in *C. chinense* BG-3821 during compatible and incompatible interaction with *Pepper golden mosaic geminivirus* (PepGMV) as well as two plant-pathogen signal molecules, namely ethylene and jasmonic acid. It was observed that *CchGLP* was induced at least from 12 h to 48 h in inoculated leaves post-inoculation or application, either by PepGMV, PHYVV, salicylic acid or ethylene (Figure 5B,C). *CchGLP* induction was also observed in newly emerged systemically geminivirus-infected leaves 15 days post-inoculation (Figure 5B). On the other hand, neither *P. capsici* infection nor jasmonic acid application induced *CchGLP* expression in *C. chinense* BG-3821 (Figure 5B,C). Compatible interaction in a geminivirus-susceptible *C. chinense* accession (UX-SMH-55) did not induce *CchGLP* when inoculated neither with PepGMV, PHYVV, *P. capsici* nor Et, JA or SA applications (not shown).

Several lines of evidence shows that GLPs are important in plant protection against pathogens and pests, or induced with applications of chemicals such as salicylic acid, ethylene or hydrogen peroxide [20–22]. A previous report showed that *CchGLP* was induced in incompatible interactions between *C. chinense* BG-3821 and PHYVV, *Xanthomonas campestris* pv. vesicatoria and not in a compatible interaction with oomycete *Phytophthora capsici* [11]. In this study, it was shown that *CchGLP* was also induced in incompatible, but not in compatible, interactions with another geminivirus affecting pepper in Mexico (PepGMV) in *C. chinense* accessions. The phenotype of susceptibility to geminiviruses in *C. chinense* UX-SMH-55 evaluated, might, at least partially, be explained by the absence of *CchGLP* induction in all infections and plant signal molecules applications evaluated. Defense response genes are also uninduced in other susceptible plants as showed elsewhere [2,11,12]. The fact that the *CchGLP* gene was not detected in the genome of the geminivirus-susceptible *C. annuum* cv. “S. Anaheim”, suggest either a low homology with this gene or absence in the genome of this pepper species, as occurred with other genes previously identified in *C. chinense* BG-3821 [12]. Moreover, *CchGLP* was also demonstrated to be induced by SA and Et applications in the absence of pathogens [11]. In this study, it was demonstrated that Et but not JA induced local and systemic *CchGLP* gene expression in the absence of pathogens. The SA and JA signaling pathways are mutually antagonistic, whereas SA and Et pathways appear to interact [23,24]. Further studies are necessary to determine the role of this signal routes in this plant-microbe interaction.

## 3. Experimental Section

### 3.1. *CchGLP* Cloning for Protein Expression in E. coli

Based upon the *CchGLP* sequence (accession number DQ677335), two oligonucleotides to amplify the *CchGLP* open reading frame without signal peptide were designed. Oligonucleotide sequences included restriction sites for *Msc* I and *Xho* I enzymes in the forward and reverse primers, respectively. The sequence of these primers (restriction sites are written in lower case) was as follows: (Forward) 5′- *tggcca*GCTGTTCAAGATTTCTGCGTCGC-3′, (Reverse) 5′-gagctcCTTAATCGTAGCTTCATCAAGG-3′. PCR reaction mix contained the following components: 0.75 μL each dNTPs (2.5 mM), 2 μL of oligonucleotides (50 ng/mL), 0.5 μL of *Taq* DNA polymerase (6 U/μL), 1 μL of DNA from *CchGLP* cloned in PCR^TM^ 4-TOPO^R^ (Invitrogen) in a reaction volume of 50 μL. PCR conditions to amplify the *CchGLP* gene were: 94 °C, 1 min; 51 °C, 1 min and 72 °C, 2 min for 35 cycles. PCR products were visualized on 1.5% agarose gels using a digital image system (1D Image Analysis Software, version 3.02; Kodak Digital System, Rochester, NY, USA). This amplified product (containing *Msc* I and *Xho* I restriction sites) was purified from agarose gel using a gene clean commercial kit (QIAGEN, Mexico, DF, Mexico) and cloned into PCR^TM^ 4-TOPO^R^. This construct was digested with the aforementioned enzymes and the *CchGLP* fragment was purified again from agarose gel. The pET22b (+) protein expression vector (Novagen), was digested with *Msc* I and *Xho* I and then ligated with the *CchGLP* fragment using T4 ligase (Promega) for 3 h at 16 °C. The resulting construct was named as pET22b(+)-*CchGLP*, and further evaluated using protein expression and enzymatic activity assays.

### 3.2. *CchGLP* Production in E. coli

A BL21 *E. coli* strain was transformed with pET22b (+)-*CchGLP*. Transformed cells were grown overnight at 32 °C in liquid Basal Salts-Glucose (BSG) medium [25]. Then, a 1 mL aliquot was inoculated into a flask with 100 mL of BSG medium with ampicillin (50 mg/mL) and 1 mM of IPTG, and grown until an OD 0.6 of 32 °C was reached. Aliquots of 5 mL of this medium were evaluated from 3 to 24 h for GLP protein expression. Samples were centrifuged at 10,000 rpm for 10 min and then resuspended in 500 μL saline phosphate buffer (SPB) [26]. Cells were disrupted using a sonicator (Ultrasonic processor) with an amplitude of 30% and 9.9 s pulses during 2 min, then centrifuged at 10,000 rpm for 10 min. Soluble fraction was separated and resuspended in SPB. Protein quantification was carried out using the protein assay kit (Bio-Rad) [27]. Proteins were separated on SDS-PAGE gels (gradient from 4–20%) and stained with Coomasie blue. The molecular size marker used was Benchmark^TM^ (Invitrogen). *CchGLP* protein purification under native conditions was carried out using affinity chromatography (ProBond, Invitrogen) following the manufacturer’s recommendations and further evaluated by Western blot analysis using Anti-His (*c*-term)-AP antibodies (Invitrogen).

### 3.3. Determination of Enzymatic Activity

Assays for OXO activity were carried out essentially as described in Dumas *et al.* [28]; the positive control was a commercial oxalate oxidase of barley (SIGMA-Aldrich). SOD activity was determined using the procedure reported by Beauchamp and Fridovich [29]. The gel was incubated in phosphate buffer pH 7.5 containing 2.5 mM of NBT, then the gel was rinsed in distilled water. Afterward, the gel was incubated in phosphate buffer containing 28 μM of Riboflavine and 28 mM TEMED for 20 min, then rinsed with distilled water and placed on a soft light source. Determination of SOD type was carried out including inhibitory concentrations of either H_2_O_2_ or KCN when evaluating Fe-SOD or Cu-Zn-SOD, respectively [19]. Commercial Fe-SOD from *E. coli* strain and bovine erythrocyte Cu-Zn-SOD were used as experimental controls (Grupo Nutramex, Mexico, DF, Mexico).

### 3.4. Virus and Oomycete Inoculation and Plant Growth Regulators Applications

Geminivirus-resistant (*C. chinense* line BG-3821) and susceptible (*C. chinense* line UX-SMH-55) plants were inoculated with 2 μg of cloned viral DNA components (cloned in SK+ bluescript plasmid, Stratagene, La Jolla, CA, USA) using a biolistic procedure with a particle delivery system (Model PDS 1000, Dupont, Wilmington, DE, USA) as reported [30]. Both pepper lines were previously identified and characterized in our group based on resistance or susceptibility to geminivirus infections [30]. Before bombardments, PHYVV components A and B, and component B of PepGMV were excised from bluescript plasmid using *Hin*dIII. Component A of *PepGMV* was excised from the plasmid with the enzyme *Eco*RI in order to increase the percentage of plants infected using the biolistic procedure [11,12,30]. Plants were inoculated at the 4 leaf-stage and maintained in a greenhouse at 26–28 °C as reported [30]. Inoculation of *P. capsici* was performed according to Barrera-Pacheco *et al*. [11]. In all cases, newly emerged uninoculated leaves were sampled at 15 days post-inoculation to analyze systemic *CchGLP* gene induction. As controls, plants were mock-inoculated with the plasmid bluescript or potato dextrose agar blocks. PHYVV and *P. capsici* inoculations were used as positive or negative controls, respectively, for *CchGLP* induction in *C. chinense* BG-3821 plants based on previous reports [11,12,30]. Applications of ethylene, jasmonic acid and salicylic acid were according to Barrera-Pacheco *et al*. [11]. Mock-inoculated plants for these treatments used water applications only.

### 3.5. Hybridization Analysis

The copy number of *CchGLP* in *C. chinense* BG-3821 was determined using standard Southern blotting protocols [26]. Gene induction studies of *CchGLP* in *C. chinense* BG-3821 and UX-SMH-55 were carried out by Northern blot (slot blot) as described in Barrera-Pacheco *et al*. [11]. A DNA fragment consisting of the complete ORF of *CchGLP* was used as a probe in both assays.

### 3.6. Analysis of Genomic Sequence of *CchGLP* Structural Region

Total genomic DNA was extracted following the procedure of Anaya-López *et al*. [12]. This DNA was used as a template for *CchGLP* structural region amplification using the specific oligonucleotides aforementioned in Section 3.1. The PCR amplified fragment was directly sequenced and compared to that from *CchGLP* cDNA, using bioinformatic tools (see below).

### 3.7. Bioinformatic Analysis

Analysis of GLP amino acid sequences was carried out using Lasergene 8.0 software (DNAstar). A phylogenetic tree was generated using the MegAlign tool of Lasergene 8.0. Accession numbers of GLP amino acid sequences evaluated in the tree are: *C chinense* (ABG76199), *A. thaliana* (NP_177405), Auxin binding protein from *Solanum nigrum* (ADW66138), GLP 24 K from *Nicotiana tabacum* (BAC77634), *GLP* from *Nicotiana tabacum* (BAH15357), Auxin binding protein from *Solanum nigrum* (ADW66150) and auxin binding protein 19a from *Prunus persica* (Q9ZRA4). Prediction of signal peptide and glycosylation sites was performed using ExPASy proteomics server tools [31].

## 4. Conclusions

The *CchGLP* gene encodes a germin-like protein with Mn-superoxide dismutase activity. This gene is induced during incompatible interactions and applications of plant defense inducer molecules such as SA and Et. Plant defense in response to microbial attack is regulated through a complex network of signaling pathways that involve three signaling molecules: SA, JA and Et. Taken together, our results support a possible role for *CchGLP* in pathogen resistance, and likely with SA and Et signal pathways involved in these events.
